# Laryngology and Rhinology

**Published:** 1898-12

**Authors:** Barclay J. Baron


### LARYNGOLOGY AND RHINOLOGY.

Dr. H. Curtis of New York read a paper at the Edinburgh Meeting of the British Medical Association on the treatment of **singers' laryngitis**,[1] in which he pointed out the importance of differentiating between the laryngitis of singers and of other people. In the former attrition is the most frequent cause. A bad method of attack, especially the *coup de glotte*, is the initial cause of trouble, which may, if neglected, go on to thickening of the membrane and the formation of nodules. For the relief of this affection Dr. Curtis suggests certain vocal exercises, which would not permit the vocal cords to touch during the attack in such a manner that attrition becomes possible. The daily practice of these exercises relieved the cords of the inflammation due to attrition and of congestion due to bad methods, and nodules disappeared without surgical interference.

Dr. Yonge read a paper on the treatment of **dysphagia in laryngeal tuberculosis**.[2] Of fifteen drugs which were tested—5 per cent. of cocaine being taken as the standard,—the following were found to be generally effective: Cocaine, antipyrin, eucaine, orthoform, carbolic acid, guaiacol, ice, morphia (with or without iodoform), and paramonochlorphenol. In the presence of ulceration, any of the above may be applied; but if it is absent, only cocaine, antipyrin, eucaine, carbolic acid, and

[1] *Brit. M. J.*, 1898, ii. 1231; abstract in *J. Laryngol.*, 1898, xiii. 459.
[2] Abstracts in *Brit. M. J.*, 1898, ii. 1250; *J. Laryngol.*, 1898, xiii. 460.

ice are available. With perichondritis antipyrin has certain advantages, and it may be mixed with cocaine if the latter has to be used for long periods. Cocaine and carbolic acid, and iced solutions of cocaine (5 per cent.), appear to be better than using the solution of ordinary temperature. Morphia and iodoform in ulcerated cases gave modified relief for several hours.

Orthoform when applied to a cleansed ulcer produced relief in a few minutes, lasting some hours; and this drug from its non-toxicity, coupled with its lung-action, possesses decided advantages over cocaine in the upper air-passages. Guaiacol (with or without menthol), eucaine (A), and solutions of paramonochlorphenol in glycerine, are all helpful, the latter producing decided anæsthesia after rather severe smarting, and it has a curative action like submucous injections of guaiacol. The prone position of Wolfenden, the œsophageal tube, and rectal feeding were all mentioned.

Dr. Mygind suggests the use of antidiphtheritic serum in the treatment of **ozæna.**[1] The use of this drug in ozæna is based on the fact that it has the power of detaching membrane from the throat in cases of diphtheria. Ten cases of genuine ozæna were treated, seven being young soldiers and three young girls. Ten cubic centimetres for adults and five for children were employed, increasing the dose to fifteen, and it was not repeated for eight to twelve days. The effect on the nasal mucous membrane is undoubted and rapid, the crusts being more easily detached, and mucous and muco-purulent secretion coming away with them within twenty-four hours; during the following days it becomes red in colour and swells, and is more or less covered with mucus. The treatment has its drawbacks, *e.g.* painful swelling around site of injection, different forms of skin eruption and of joint affection. Experiments show that the presence of the toxins is of no importance, but that it is the serum alone that acts, and the results of treating the disease with injections of normal serum of horses are quite as good as these mentioned.

Dr. Moure[2] suggests the following treatment of ozæna. Cleanse the nostrils, and rub them with a cotton-wool swab dipped in iodine, 2 to 5 parts, pot. iod., 4 to 6 parts, trichloracetic acid, 3 parts, glycerine, 1200 parts; or menthol, 20 to 40 parts, eucalyptus, 2 parts, ol. vaselini, 1200 parts. The secretion is then removed with a syringe, and powder containing 5 to 25 per cent. of powdered nitrate of silver is applied.

Dr. Berger[3] reports a case of ozæna completely cured by massage of the nostril with a swab covered with menthol

1 *J. Laryngol.*, 1898, xiii. 379.

2 *Rev. internat. de Rhinol., Otol. et Laryngol.*, 1897, vii. 177; abstract in *J. Laryngol.*, 1898, xiii. 246.

3 *Arch. internat. de Laryngol.* [etc.], No. 2, 1898.

solution: at first it was done every day; and then, at longer intervals, this was combined with irrigation and insufflation of boracic acid powder. The treatment lasted for three months. The atrophy of the turbinated bodies persisted, but all symptoms ceased.

Dr. Cohen Tervaert[1] describes a curious case of **ventricular laryngocele** in a man sixty-two years old, who was troubled with difficulty in breathing, especially marked on going upstairs. On examining the larynx, almost the whole of its entrance was seen to be covered with a bulla; the left side of the larynx was motionless, the right side had its normal mobility. On a second examination the bulla was absent, but returned on making a vocal effort and gradually reached its full size. Some smaller bullæ also made their appearance, and it looked as if the ventricle of Morgagni were enlarged. It is believed that the swelling is caused by a dilated ventricle, or by an appendix distended with air in respiration, which gets into it by a tear in the mucous membrane, the walls of the ventricle having become thin and yielding to pressure. The bulla protrudes through a hole in the wall of the ventricle, and it is proposed to remove it by means of a snare.

Dr. Phillips[2] relates details of a case of **primary epithelioma of the maxillary antrum.** The patient was fifty-eight years old, who had had an opening made into the antrum some years before because of pain, and this opening had never closed. Four months ago he noticed a growth around the opening, and this had rapidly increased and was cauliflower-like, two inches long and three-quarters of an inch broad. It extended into the antrum and bled freely on probing; there was no external swelling, and transillumination gave a dark shadow on affected side. The opening was enlarged by chisel and curette, and a mass scraped out, the hemorrhage being very free. The cavity was cleansed with perchloride solution and packed with iodoform gauze. For fourteen months after operation there has been no recurrence and there are no symptoms, although the growth was an epithelioma so far as microscopic examination can determine. The growth consisted of ordinary mucous polyp, at one part of which epithelioma had grown, practically as a malignant growth on a benign one.

Dr. Max Scheier[3] writes on the application of **Röntgen rays** in laryngology and rhinology.

(*a*) The sounding of the frontal sinus.

This has been doubted by some and affirmed by others,

[1] *Arch. internat. de Laryngol.* [etc.], No. 2, 1898. [2] *J. Laryngol.*, 1898, xiii. 325.
[3] *Arch. internat. de Laryngol.* [etc.], 1898, xi. 87; abstract in *J. Laryngol.*, 1898, xiii. 370.

although most rhinologists are able to get a probe into the sinus in a certain proportion of cases. The Röntgen rays enable us to see the sound in the sinus, and it is possible to reach the anterior and superior walls of it. In little children it is impossible to see the sinus with the rays, which accords with Zuckerkandl's observation that in the newly-born child there is no trace of sinus. In cases where apparently the sound was in the sinus, the rays showed that it was really in an ethmoidal cell; but the operation of probing it is undoubtedly possible, though it varies greatly in its ease or difficulty.

(*b*) In the study of the physiology of the voice and speech. The instruments must be quite perfect and the observations carried out in darkness, even the tubes being covered with black cloth and placed as near as possible to the face, care being taken not to hurt the face or the hair by too long exposure, and it is wise to cover the latter with lanoline. The lips and their movements, the tongue and its movements in the formation of letters, and the alterations of position of the palate can all be watched. The larynx, and especially spots of ossification in its cartilages, can readily be seen, and it is possible to distinguish the cricoid from the thyroid. The epiglottis cannot be seen in the living subject.

The vexed question of breathing in singers is set at rest by the X-rays, because it can be seen that in diaphragmatic—*i.e.* singers'—breathing, the diaphragm is depressed more than 8 or 10 centimetres in deep breathing in the axillary line and gradually and slowly rises in expiration. In clavicular breathing, on the other hand, it is not so deeply depressed in inspiration, and rises by jerks on expiration.

(*c*) Physiology of deglutition.

It is better to have subjects who have lost their molar teeth for this examination. The morsels swallowed should have bismuth mixed with them to render them opaque, and of liquids the best to use is "*Eau d'or de Dantzig*," as we see the gold flakes. All the various movements of mastication can be well seen, also those of the palate and larynx in swallowing.

The whole paper is most valuable, and should be read by those interested in the matters of which it treats.

BARCLAY J. BARON.

